# Mineral metabolism and outcomes in chronic kidney disease stage 2–4 patients

**DOI:** 10.1186/1471-2369-14-14

**Published:** 2013-01-16

**Authors:** Kamonwan Chartsrisak, Kotcharat Vipattawat, Montira Assanatham, Arkom Nongnuch, Atiporn Ingsathit, Somnuek Domrongkitchaiporn, Vasant Sumethkul, Sinee Distha-Banchong

**Affiliations:** 1 Division of Nephrology, Department of Medicine, Faculty of Medicine, Ramathibodi Hospital, Mahidol University, Bangkok, 10400, Thailand

**Keywords:** CKD, PTH, Vitamin D, Dialysis, ESRD, Thailand

## Abstract

**Background:**

Marked hyperphosphatemia, hyperparathyroidism and 25-hydroxyvitamin D deficiency are associated with mortality in dialysis patients. Such data in chronic kidney disease stage 2–4 population are limited. It has been suggested that high-normal serum phosphate predicts worse renal and patient outcomes. The data regarding parathyroid hormone and outcomes in this population is limited. The present study examined mineral metabolism and its association with the development of end-stage renal disease and mortality in stage 2–4 chronic kidney disease patients.

**Methods:**

This is a prospective cohort study that included 466 non-dialysis chronic kidney disease stage 2–4 patients. Mineral parameters were obtained at the time of enrollment and the patients were followed prospectively for 25 (1–44) months or until they reached the endpoints of end-stage renal disease or mortality.

**Results:**

Hyperparathyroidism and 25-hydroxyvitamin D deficiency began to occur in the early stages of chronic kidney disease, whereas significant hyperphosphatemia only developed in the later stages. High-normal and mildly elevated serum phosphate (>4.2 mg/dL) predicted the composite outcome of end-stage renal disease or mortality after adjustments for cardiovascular risk factors, chronic kidney disease stage and other mineral parameters. Parathyroid hormone levels above the upper limit of normal (>65 pg/mL) predicted the future development of end-stage renal disease and the composite outcome of end-stage renal disease or mortality after adjustments. 25-hydroxyvitamin D deficiency (<15 ng/mL) was also associated with worse outcomes.

**Conclusions:**

In chronic kidney disease, hyperparathyroidism developed prior to significant hyperphosphatemia confirming the presence phosphate retention early in the course of chronic kidney disease. High-normal serum phosphate and mildly elevated parathyroid hormone levels predicted worse renal and patient outcomes. This data emphasizes the need for early intervention in the care of chronic kidney disease stage 2–4 patients.

## Background

Chronic kidney disease (CKD) is associated with an increased risk of mortality [1]. As kidney function deteriorates, abnormalities of mineral metabolism began to develop. Hyperphosphatemia, hyperparathyroidism and vitamin D deficiency have been shown to predict all-cause and cardiovascular mortality in dialysis patients [2,3]. Derangements of mineral metabolism began to occur in the early stages of chronic kidney disease [4]. Despite the presence of phosphate retention in this early period, hyperphosphatemia does not develop until later stages owing to an increase in parathyroid hormone (PTH) and fibroblast growth factor 23 (FGF-23) whose actions result in augmented urinary phosphate excretion [5]. Previous studies have also suggested that, in less advanced stages of CKD, an increase in serum phosphate albeit within the normal range in association with an increase in FGF-23 predicted the development of cardiovascular events and mortality [6-8]. This data emphasizes the importance of phosphate retention on patient outcomes. Limited data is available regarding the relationship between PTH and outcomes in CKD stages 2–4. The aim of the present study was to examine the associations between mineral parameters including serum phosphate, PTH and vitamin D and outcomes of end-stage renal disease (ESRD) and mortality in CKD stage 2–4 patients.

## Methods

### Patients

This study was approved by the ethical committee for research involving human subjects of the Faculty of Medicine, Ramathibodi Hospital, Mahidol University and was conducted according to the Declaration of Helsinki. Informed consent was obtained from all participants. Non-dialysis CKD patients with serum creatinine >=1.1 mg/dL in females, and >=1.2 mg/dL in males, were recruited during routine follow-up visits to the outpatient clinic of Ramathibodi Hospital, between May 2008 to December 2010. Patients with acute illnesses or acute kidney injury were excluded.

Medical chart review was performed to collect data on baseline demographics and characteristics. Cardiovascular disease (CVD) was defined by a history of coronary artery disease (myocardial infarction, unstable angina, positive coronary angiography or abnormal myocardial perfusion scan), cerebrovascular disease or peripheral arterial disease. Diabetes mellitus (DM) was defined according to the WHO criteria or the use of hypoglycemic agents. Dyslipidemia was defined as total cholesterol >= 240 mg/dL, LDL >=130 mg/dL, triglycerides >=200 mg/dL, or by the use of statin. The history of calcium intake was defined as receiving oral calcium tablets with elemental calcium >=500 mg per day for at least 3 months prior to the recruitment. The history of nutritional vitamin D intake was defined as receiving a cumulative dose of >=5,000 IU of ergocalciferol or cholecalciferol per month for at least 3 months prior to the recruitment. The history of active vitamin D intake was defined as receiving calcitriol or alfacalcidol at a minimum dosage of 0.25 μg 3 times per week for at least 3 months prior to the recruitment. None of the patients received non-calcium-containing phosphate binders or calcimimetics because the availabilities of these drugs in Thailand were limited.

### Laboratory data

Laboratory data including serum calcium, phosphate, albumin, cholesterol, alkaline phosphatase (ALP), blood urea nitrogen (BUN), creatinine (Cr), intact parathyroid hormone (PTH), 25-hydroxyvitamin D (25-OH-D) and spot urine protein creatinine ratio (UPCR) were obtained at the time of enrollment. Blood and urine samples were analyzed using a Dade Behring Dimension RxL chemical analyzer (Siemens, Germany). Intact PTH was determined by an immunoradiometric assay (ELISA-PTH, Cisbio International, France). 25-OH-D was determined by a chemiluminescent immunoassay. Serum calcium was corrected based on the following equation: corrected calcium = serum calcium + [(40 – serum albumin) ÷ (10 × 0.8)]. Estimated GFR was calculated using a modified MDRD formula validated for the Thai population: eGFR = (375.5 x Cr^(−0.848)^ × Age^(−0.364)^) × 0.712 if female [9].

### Outcomes

The study commenced at the time of enrollment when all laboratory data were obtained. Patients were followed prospectively until they reached ESRD, mortality or the end of January 2012. Outcomes were time to death-censored ESRD or time to the composite outcome of ESRD or all-cause mortality, whichever came first. Those who were lost to follow-up were contacted by phone and asked whether they were still alive and remained free of dialysis. The survival data of the patients who could not be reached by phone were cross-referenced with the national civil registration database and therefore, the mortality data was available in all patients. Eleven patients who did not have a follow-up renal function were presumed to be free of dialysis.

### Statistical analysis

Results are presented as mean ± SD unless specified otherwise. One-way ANOVA or Kruskal-Wallis test were used to compare the differences among groups of continuous data. Chi-square test was applied to test the differences among groups of categorical data. The significance of trends of multiple groups was analyzed by linear-by-linear association in a Chi-square test. The relationship between two continuous variables was evaluated by a Pearson’s correlation. Non-normal distribution data were log-transformed prior to analyses. Cox proportional hazards regression was used to determine the associations between mineral parameters and outcomes. Cox models were adjusted for age, sex, DM, BMI, serum albumin, eGFR (>=45 or <45 mL/min/1.73 m^2^) and mineral parameters at baseline. Variables were checked for multicolinearity prior to entering into the multivariate models. All computations were performed using SPSS version 17.0 software (SPSS, Chicago, IL). A p-value of less than 0.05 was considered statistically significant.

## Results

### Baseline characteristics and mineral parameters

**Figure 1:**
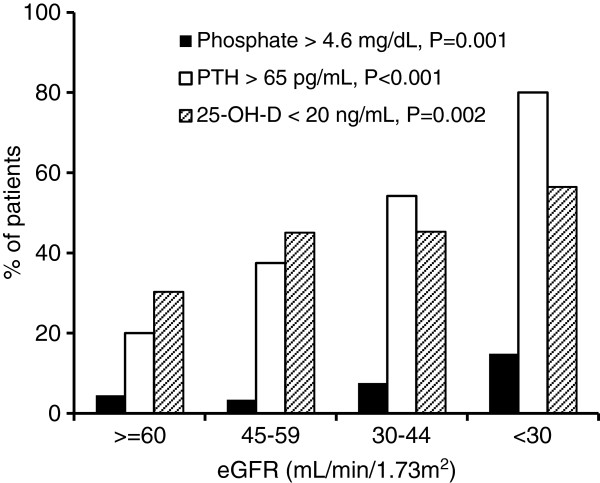
**Percentage of patients with hyperphosphatemia, hyperparathyroidism and 25-hydroxyvitamin D deficiency according to renal function. ***P*-values represent the significance of trend for each mineral parameter.

A total of 466 patients were enrolled in the study. The median follow-up time was 24.6 (range 1–44) months. Baseline demographics and laboratory data of all patients and classification according to the eGFR are shown in Table 1. As renal function deteriorated, serum phosphate and PTH increased significantly, whereas serum calcium was largely unchanged. With rising serum phosphate and PTH, more patients were prescribed oral calcium and active vitamin D. Despite the decrease in 25-OH-D levels with worsening renal function, there was no significant change in the number of patients who received nutritional vitamin D supplements. In addition to the eGFR, serum phosphate was positively correlated with serum calcium (*r*=0.177, *P*<0.001) and PTH (*r*=0.176, *P*<0.001) but negatively correlated with 25-OH-D (*r*=−0.157, *P*<0.001). An inverse relationship between PTH and serum calcium (*r*=−0.274, *P*<0.001) as well as 25-OH-D (*r*=−0.216, *P*<0.001) was observed. 25-OH-D displayed a strong negative association with UPCR (*r*=−0.354, *P*<0.001) and correlated positively with serum albumin (*r*=0.245, *P*<0.001). Figure 1 illustrated the prevalence of hyperphosphatemia, hyperparathyroidism and 25-OH-D deficiency according to the eGFR. Significant hyperparathyroidism and 25-OH-D deficiency occurred earlier in the course of CKD, but significant hyperphosphatemia only developed in the later period. Overall in this population of CKD stage 2–4 patients, 36 (7.7%) patients had hyperphosphatemia, 229 (49.1%) had hyperparathyroidism and 213 (45.7%) were vitamin D deficient.

**Table 1:** Baseline demographics and laboratory data of all patients and classification according to renal function

	**eGFR (mL/min/1.73m**^**2**^**)**					
**Parameters**	**All Patients (n=466)**	**>=60 (n=67)**	**45-59 (n=123)**	**30-44 (n=161)**	**<=29 (n=115)**	***P-value***^***a***^
Age (yrs)	65±13.2	61.2±15.1	62.6±13.7	66.1±12.3	68±11.6	<0.001
Male Sex (n/%)	261 (56)	60 (89.6)	90 (73.2)	75 (46.6)	36 (31.3)	<0.001
BMI (kg/m^2^)	25.4±4.7	25±3.9	26.6±5.2	25±4.5	24.9±4.6	0.16
Hypertension (n/%)	422 (90.6)	58 (86.6)	115 (93.5)	147 (91.3)	102 (88.7)	0.948
Dyslipidemia (n/%)	324 (69.5)	49 (73.1)	85 (69.1)	114 (70.8)	76 (66.1)	0.404
DM (n/%)	263 (56.4)	28 (41.8)	65 (52.8)	102 (63.4)	68 (59.1)	0.011
CVD (n/%)	104 (22.3)	9 (13.4)	25 (20.3)	42 (26.1)	28 (24.3)	0.967
Calcium intake (n/%)	133 (28.5)	10 (14.9)	22 (17.9)	45 (28)	56 (48.7)	<0.001
Nutritional vitamin D (n/%)	49 (10.5)	7 (10.4)	11 (8.9)	20 (12.4)	11 (9.6)	0.972
Active vitamin D (n/%)	18 (3.9)	0 (0)	1 (0.8)	4 (2.5)	13 (11.3)	<0.001
*Laboratory data*						
Calcium (mg/dL)^b^	9.3±0.5	9.19±0.53	9.27±0.43	9.32±0.44	9.28±0.55	0.195
Phosphate (mg/dL)	3.8±0.6	3.45±0.51	3.58±0.55	3.81±0.56	4.12±0.71	<0.001
Alkaline Phosphatase (U/L)	73.4±28.6	65.5±23.6	67.8±25	78.2±27.9	79.2±34.9	0.034
PTH (pg/mL)^c^	66 (43–105)	40 (29–61)	58 (38–74)	70 (48–103)	117 (71–173)	<0.001
25-OH-D (ng/mL)	22±9.1	24.6±9.2	22.7±10.3	22.1±8.3	19.8±8.3	<0.001
Albumin (g/L)	37.5±5.2	38.1±6.6	39.2±4.3	37.1±5.2	36±4.8	<0.001
Cholesterol (mg/dL)	192±56	205±74	194±55	188±57	189±41	0.301
BUN (mg/dL)	29.7±14.6	16.1±4.7	22.4±6.8	29.6±9.7	45.1±16.2	<0.001
Creatinine (mg/dL)	2.25±1.21	1.23±0.16	1.62±0.34	2.12±0.49	3.68±1.53	<0.001
eGFR (mL/min/1.73 m^2^)	42.3±16.4	70.2±9.6	51.9±4.4	37.1±4.1	22.1±5.2	<0.001
UPCR (mg/g)^c,d^	0.67 (0.21-2.43)	0.24 (0.11-0.88)	0.35 (0.12-1.72)	0.58 (0.24-1.89)	1.78 (0.77-3.86)	<0.001

^a^*P-value* for trend from eGFR>=60 to <=29, ^b^corrected calcium, ^c^median (IQR), ^d^spot urine protein/creatinine ratio.

### Mineral parameters and outcomes

**Figure 2:**
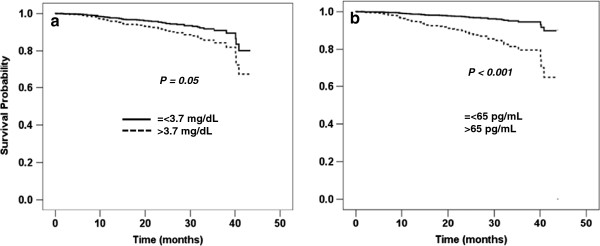
**Adjusted survival curves of mineral parameters and the outcome of ESRD. a**) serum phosphate and **b**) PTH. Adjusted for age, sex, DM, BMI, serum albumin and eGFR (>=45 or <45 mL/min/1.73 m^2^).

**Figure 3:**
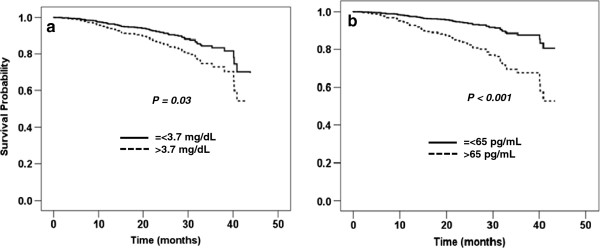
**Adjusted survival curves of mineral parameters and the composite outcome of ESRD or mortality. a**) serum phosphate and **b**) PTH. Adjusted for age, sex, DM, BMI, serum albumin and eGFR (>=45 or <45 mL/min/1.73 m^2^).

At the end of the follow-up period, ESRD occurred in 74 (15.9%) patients, death in 40 (8.6%) patients, and both ESRD and death in 6 (1.3%) patients. Outcomes in the present study were time to death-censored ESRD or time to the composite outcome of ESRD or all-cause mortality. To analyze whether any of the baseline mineral parameters were associated with outcomes, serum calcium, phosphate, PTH and 25-OH-D were divided into tertiles or quartiles. In univariable Cox proportional hazards models, serum calcium, oral calcium or vitamin D intake did not show significant associations with the outcomes. Serum phosphate greater than 3.7 mg/dL was associated with the development of ESRD in an unadjusted model (Table 2). After adjustments for eGFR (>=45 or <45 mL/min/1.73 m^2^) and CVD risk factors including age, sex, DM, BMI and serum albumin (Model 1), only the highest tertile of serum phosphate (> 4.2 mg/dL) predicted future ESRD. The relationship between serum phosphate and the outcome was lost after the model was further adjusted for PTH and 25-OH-D (Model 2). High-normal PTH levels (42–65 pg/mL) were associated with the development of ESRD in an unadjusted model. After adjustments for eGFR and CVD risk factors and mineral parameters including phosphate and 25-OH-D, only PTH levels above the upper limit of normal (>65 pg/mL) were associated with ESRD. 25-OH-D levels less than 15 ng/mL predicted the future development of ESRD. The relationship between low 25-OH-D levels and ESRD remained significant after adjustments for eGFR, CVD risk factors and serum phosphate. PTH and 25-OH-D levels were highly correlated; therefore, PTH was excluded from the multivariate model. With the composite outcome of ESRD or mortality, the highest tertile of serum phosphate was associated with the outcome in all models. Results similar to those obtained with the outcome of ESRD were observed with PTH and 25-OH-D (Table 3). When only mortality event was used in survival analyses, the relationship between mineral parameters and the outcome was not found. Adjusted survival curves according to the median serum phosphate and PTH are shown in Figures 2 and 3. Serum phosphate greater than 3.7 mg/dL and PTH levels higher than 65 pg/mL were associated with the progression to ESRD and the composite outcome of ESRD or mortality after adjustments for age, sex, DM, BMI, serum albumin and eGFR (>=45 or <45 mL/min/1.73 m^2^).

**Table 2:** Hazard ratios of ESRD

**Parameters**	**Unadjusted**	**Model 1**^**a**^	**Model 2**^**b**^
Phosphate^d^ (mg/dL)			
0-3.7	1 (reference)	1 (reference)	1 (reference)
3.7-4.2	2.04 (1.13-3.7)*	1.53 (0.77-3.03)	1.81 (0.86-3.81)
>4.2	4.56 (2.59-8.03)*	2.38 (1.21-4.68)*	1.98 (0.92-4.28)
PTH^e^ (pg/mL)			
1-42	1 (reference)	1 (reference)	1 (reference)
42-65	12.7 (1.65-97.5)*	5.73 (0.7-46.6)	5.66 (0.69-46.2)
65-105	13.2 (1.74-99.9)*	8.6 (1.09-67.7)*	8.77 (1.12-68.8)*
>105	51.4 (7.08-374)*	20.8 (2.75-158)*	16.5 (2.18-125)*
25-OH-D^f^ (ng/mL)			
>30	1 (reference)	1 (reference)	1 (reference)
15-30	1.84 (0.78-4.34)	3.64 (0.86-15.4)	3.33 (0.78-14.1)
<15	4.07 (1.67-9.92)*	6.82 (1.52-30.6)*	5.73 (1.27-26)*

^a^adjusted for age, sex, DM, BMI, serum albumin and eGFR (>=45 or <45 mL/min/1.73 m^2^), ^b^Model 1 further adjusted for other mineral parameters in this table. ^d^normal values 2.5-4.6 mg/dL. ^e^normal values 15–65 pg/mL, ^f^normal values >=20 ng/mL. *P<0.05.

**Table 3:** Hazard ratios of the composite outcome of ESRD or mortality

**Parameters**	**Unadjusted**	**Model 1**^**a**^	**Model 2**^**b**^
Phosphate^d^ (mg/dL)			
0-3.7	1 (reference)	1 (reference)	1 (reference)
3.7-4.2	1.67 (1.03-2.7)*	1.45 (0.82-2.55)	1.5 (0.82-2.74)
>4.2	3.51 (2.21-5.57)*	2.31 (1.31-4.05)*	2.01 (1.08-3.74)*
PTH^e^ (pg/mL)			
1-42	1 (reference)	1 (reference)	1 (reference)
42-65	2.22 (1.01-4.92)*	2.28 (0.79-6.56)	2.3 (0.8-6.63)
65-105	2.13 (0.98-4.64)	3.38 (1.22-9.39)*	3.54 (1.27-9.87)*
>105	7.16 (3.52-14.5)*	6.81 (2.52-18.4)*	6.04 (2.23-16.4)*
25-OH-D^f^ (ng/mL)			
>30	1 (reference)	1 (reference)	1 (reference)
15-30	1.27 (0.7-2.31)	2.25 (0.88-5.71)	2.13 (0.0.84-5.43)
<15	2.28 (1.19-4.37)*	3.27 (1.19-8.96)*	2.85 (1.03-7.84)*

^a^adjusted for age, sex, DM, BMI, serum albumin and eGFR (>=45 or <45 mL/min/1.73 m^2^), ^b^Model 1 further adjusted for other mineral parameters in this table. ^d^normal values 2.5-4.6 mg/dL. ^e^normal values 15–65 pg/mL, ^f^normal values >=20 ng/mL.*P<0.05.

## Discussion

The present study evaluated mineral metabolism and the associations between mineral parameters and outcomes of ESRD and mortality in CKD stage 2–4 patients. High prevalence of hyperparathyroidism and 25-OH-D deficiency was observed in the early stages of CKD, whereas significant hyperphosphatemia only developed in the later stages. High-normal and mildly elevated serum phosphate predicted the composite outcome of ESRD or mortality. Mildly elevated PTH levels above the upper limit of normal were associated with the development of ESRD and the composite outcome of ESRD or mortality.

Hyperphosphatemia is a well-known risk factor for mortality in ESRD patients [2,10-13]. Less data is available in the non-dialysis CKD population. In early stages of CKD, serum phosphate levels are mostly within the normal range owing to augmented renal phosphate excretion as a result of the release of at least 2 phosphaturic hormones: PTH and FGF-23 [5,14]. In the present study, CKD patients developed hyperparathyroidism starting from stage 2. By the time they reached stage 4, hyperparathyroidism occurred in almost 80% of the patients. Previous studies have also demonstrated the appearance of hyperparathyroidism as early as eGFR 70–80 mL/min, whereas hyperphosphatemia only became pronounced once eGFR fell below 40–50 mL/min [4,5,15].

In the present study, patients who belonged to the highest tertile of serum phosphate (>4.2 mg/dL) had an increased risk of ESRD and mortality. Since only 7.7% of the population were hyperphosphatemic, most patients who belonged to this highest tertile of serum phosphate actually had serum phosphate within the normal range. Moreover, when the patients were divided according to the median serum phosphate of 3.7 mg/dL, those with serum phosphate greater than 3.7 mg/dL had a greater risk of progression to ESRD and mortality. This data suggests that a state of phosphate retention that began long before hyperphosphatemia posed a significant risk to patient outcomes. This finding is in agreement with the results from prior studies. Kestenbaum et al. reported that serum phosphate higher than 3.5 mg/dL was associated with an increase in all-cause mortality [6]. Bellasi et al. observed an association between serum phosphate >=4.3 mg/dL and the risk of starting dialysis or dying [16]. Higher serum phosphate concentrations within the normal range were also associated with a more rapid decline in renal function and a progression to ESRD [8]. The evidence of a relationship between increasing serum phosphate and adverse patient outcomes has been observed in subjects without CKD. In patients with CVD, serum phosphate 3.5 mg/dL or higher was associated with an increased risk of death compared to serum phosphate lower than 3.5 mg/dL [17]. In type 2 diabetes, serum phosphate greater than 3.9 mg/dL was associated an increase in cardiovascular mortality [18]. In community dwelling non-CKD, non-CVD adults, higher serum phosphate increased CVD risks after a follow-up period of over 10 years [19,20]. Whether an association between phosphate load and worse outcomes is the result of a direct or indirect effect of phosphate will require further study. Previous studies have demonstrated that a high phosphate environment could induce endothelial cell apoptosis in vitro, and oral phosphate load in healthy volunteers resulted in a decrease in flow-mediated arterial dilatation indicating an impairment of endothelial function [21,22]. Higher serum phosphate levels in non-dialysis CKD patients were found to be associated with increased levels of c-reactive protein and inflammatory cytokines [23]. In subjects with preserved renal function, increased serum phosphate was associated with the presence of calcified coronary atherosclerotic plaque [24].

As mentioned earlier, in the course of CKD, PTH and FGF-23 levels become elevated prior to the development of hyperphosphatemia. An increase in FGF-23 levels has been shown to predict all-cause and cardiovascular mortality in both dialysis and non-dialysis CKD populations [25,26]. Limited data is available regarding the relationship between PTH and outcomes especially in the early stages of CKD. In ESRD, markedly elevated PTH concentrations (>450-600 pg/mL) predicted worse outcomes, whereas a more modest increase did not [2,3,11,13]. In the present study, an increase in PTH levels just above the upper limit of normal (>65 pg/mL) predicted the future development of ESRD and mortality independent of CVD risks and other mineral parameters. There was only one other study in male non-dialysis CKD patients that reported an association between mildly elevated PTH levels and the risk of mortality [27]. While the data on FGF-23 and renal and cardiovascular outcomes continue to accumulate, the link between excess PTH and adverse patient outcomes beyond the musculoskeletal system has not been emphasized [26,28-30]. It has been suggested that, in general population, an increase in PTH above the normal limit in association with low 25-OH-D levels predicted the development of sudden cardiac death 14 years later [31]. Serum PTH also correlated with an increase in systolic and diastolic blood pressure, and patients with mild primary hyperparathyroidism exhibited increased arterial stiffness [32,33]. Heightened activity of renin-angiotensin-aldosterone system was proposed to be the link between PTH and adverse patient outcomes [34]. The relationship between 25-OH-D deficiency and worse renal and patient outcomes in CKD patients not yet on dialysis has been documented previously [35-37]. Similar results are also observed in the present study.

The present study has some limitations. The relatively low number of patients and low event rates may impact the study models. FGF-23 levels were not determined precluding an assessment of the effect of FGF-23 on outcomes. As renal function deteriorates, mineral parameters change overtime and, the use of time-dependent covariates may be more appropriate in this setting. The present study is observational in nature and some laboratory data were not available during the follow-up period. Nevertheless, the outcome data including renal function was obtained in 98% of the patients and mortality data was available in all patients.

## Conclusions

In conclusion, hyperparathyroidism developed prior to significant hyperphosphatemia confirming the presence of phosphate retention in the early stages of CKD. High-normal serum phosphate and mildly elevated PTH levels predicted the future development of ESRD and mortality in CKD stage 2–4 patients.

## Competing interests

Sinee Distha-Banchong has received speaker’s honoraria from Fresinius-Kabi and Sanofi. No financial competing interest.

## Authors’ contributions

KC, AI and MA collected and analyzed the data and drafted and revised the manuscript. KV, AN, SD and VS collected the data, provided clinical experience and revised the manuscript. SDB conceptualized and designed the research, collected and analyzed the data, and drafted and revised the manuscript. All authors read and approved the final manuscript.

## Pre-publication history

The pre-publication history for this paper can be accessed here:

http://www.biomedcentral.com/1471-2369/14/14/prepub

## References

[B1] Hemmelgarn BR Manns BJ Lloyd A James MT Klarenbach S Quinn RR Wiebe N Tonelli M Alberta Kidney Disease N Relation between kidney function, proteinuria, and adverse outcomes JAMA 2010 303 5 423 429 10.1001/jama.2010.39 20124537

[B2] Block GA Klassen PS Lazarus JM Ofsthun N Lowrie EG Chertow GM Mineral metabolism, mortality, and morbidity in maintenance hemodialysis J Am Soc Nephrol 2004 15 8 2208 2218 10.1097/01.ASN.0000133041.27682.A2 15284307

[B3] Floege J Kim J Ireland E Chazot C Drueke T de Francisco A Kronenberg F Marcelli D Passlick-Deetjen J Schernthaner G Serum iPTH, calcium and phosphate, and the risk of mortality in a European haemodialysis population Nephrol Dial Transplant 2011 26 6 1948 1955 10.1093/ndt/gfq219 20466670 PMC3107766

[B4] Levin A Bakris GL Molitch M Smulders M Tian J Williams LA Andress DL Prevalence of abnormal serum vitamin D, PTH, calcium, and phosphorus in patients with chronic kidney disease: results of the study to evaluate early kidney disease Kidney Int 2007 71 1 31 38 10.1038/sj.ki.5002009 17091124

[B5] Isakova T Wahl P Vargas GS Gutierrez OM Scialla J Xie H Appleby D Nessel L Bellovich K Chen J Fibroblast growth factor 23 is elevated before parathyroid hormone and phosphate in chronic kidney disease Kidney Int 2011 79 12 1370 1378 10.1038/ki.2011.47 21389978 PMC3134393

[B6] Kestenbaum B Sampson JN Rudser KD Patterson DJ Seliger SL Young B Sherrard DJ Andress DL Serum phosphate levels and mortality risk among people with chronic kidney disease J Am Soc Nephrol 2005 16 2 520 528 10.1681/ASN.2004070602 15615819

[B7] Seiler S Reichart B Roth D Seibert E Fliser D Heine GH FGF-23 and future cardiovascular events in patients with chronic kidney disease before initiation of dialysis treatment Nephrol Dial Transplant 2010 25 12 3983 3989 10.1093/ndt/gfq309 20525642

[B8] Voormolen N Noordzij M Grootendorst DC Beetz I Sijpkens YW van Manen JG Boeschoten EW Huisman RM Krediet RT Dekker FW High plasma phosphate as a risk factor for decline in renal function and mortality in pre-dialysis patients Nephrol Dial Transplant 2007 22 10 2909 2916 10.1093/ndt/gfm286 17517792

[B9] Praditpornsilpa K Townamchai N Chaiwatanarat T Tiranathanagul K Katawatin P Susantitaphong P Trakarnvanich T Kanjanabuch T Avihingsanon Y Tungsanga K The need for robust validation for MDRD-based glomerular filtration rate estimation in various CKD populations Nephrol Dial Transplant 2011 26 9 2780 2785 10.1093/ndt/gfq815 21357214

[B10] Block GA Hulbert-Shearon TE Levin NW Port FK Association of serum phosphorus and calcium x phosphate product with mortality risk in chronic hemodialysis patients: a national study Am J Kidney Dis 1998 31 4 607 617 10.1053/ajkd.1998.v31.pm9531176 9531176

[B11] Slinin Y Foley RN Collins AJ Calcium, phosphorus, parathyroid hormone, and cardiovascular disease in hemodialysis patients: the USRDS waves 1, 3, and 4 study J Am Soc Nephrol 2005 16 6 1788 1793 10.1681/ASN.2004040275 15814832

[B12] Young EW Albert JM Satayathum S Goodkin DA Pisoni RL Akiba T Akizawa T Kurokawa K Bommer J Piera L Predictors and consequences of altered mineral metabolism: the Dialysis Outcomes and Practice Patterns Study Kidney Int 2005 67 3 1179 1187 10.1111/j.1523-1755.2005.00185.x 15698460

[B13] Covic A Kothawala P Bernal M Robbins S Chalian A Goldsmith D Systematic review of the evidence underlying the association between mineral metabolism disturbances and risk of all-cause mortality, cardiovascular mortality and cardiovascular events in chronic kidney disease Nephrol Dial Transplant 2009 24 5 1506 1523 10.1093/ndt/gfn613 19001560

[B14] Gutierrez O Isakova T Rhee E Shah A Holmes J Collerone G Juppner H Wolf M Fibroblast growth factor-23 mitigates hyperphosphatemia but accentuates calcitriol deficiency in chronic kidney disease J Am Soc Nephrol 2005 16 7 2205 2215 10.1681/ASN.2005010052 15917335

[B15] Muntner P Vupputuri S Coresh J Uribarri J Fox CS Metabolic abnormalities are present in adults with elevated serum cystatin C Kidney Int 2009 76 1 81 88 10.1038/ki.2009.76 19295502 PMC3049931

[B16] Bellasi A Mandreoli M Baldrati L Corradini M Di Nicolo P Malmusi G Santoro A Chronic kidney disease progression and outcome according to serum phosphorus in mild-to-moderate kidney dysfunction Clin J Am Soc Nephrol 2011 6 4 883 891 10.2215/CJN.07810910 21393493 PMC3069383

[B17] Tonelli M Sacks F Pfeffer M Gao Z Curhan G Cholesterol, Recurrent Events Trial I: Relation between serum phosphate level and cardiovascular event rate in people with coronary disease Circulation 2005 112 17 2627 2633 10.1161/CIRCULATIONAHA.105.553198 16246962

[B18] Chonchol M Dale R Schrier RW Estacio R Serum phosphorus and cardiovascular mortality in type 2 diabetes Am J Med 2009 122 4 380 386 10.1016/j.amjmed.2008.09.039 19332233

[B19] Dhingra R Sullivan LM Fox CS Wang TJ D'Agostino RB Sr Gaziano JM Vasan RS Relations of serum phosphorus and calcium levels to the incidence of cardiovascular disease in the community Arch Intern Med 2007 167 9 879 885 10.1001/archinte.167.9.879 17502528

[B20] Foley RN Collins AJ Ishani A Kalra PA Calcium-phosphate levels and cardiovascular disease in community-dwelling adults: the Atherosclerosis Risk in Communities (ARIC) Study Am Heart J 2008 156 3 556 563 10.1016/j.ahj.2008.05.016 18760141

[B21] Di Marco GS Hausberg M Hillebrand U Rustemeyer P Wittkowski W Lang D Pavenstadt H Increased inorganic phosphate induces human endothelial cell apoptosis in vitro Am J Physiol Renal Physiol 2008 294 6 F1381 F1387 10.1152/ajprenal.00003.2008 18385273

[B22] Shuto E Taketani Y Tanaka R Harada N Isshiki M Sato M Nashiki K Amo K Yamamoto H Higashi Y Dietary phosphorus acutely impairs endothelial function J Am Soc Nephrol 2009 20 7 1504 1512 10.1681/ASN.2008101106 19406976 PMC2709683

[B23] Navarro-Gonzalez JF Mora-Fernandez C Muros M Herrera H Garcia J Mineral metabolism and inflammation in chronic kidney disease patients: a cross-sectional study Clin J Am Soc Nephrol 2009 4 10 1646 1654 10.2215/CJN.02420409 19808245 PMC2758261

[B24] Shin S Kim KJ Chang HJ Cho I Kim YJ Choi BW Rhee Y Lim SK Yang WI Shim CY Impact of serum calcium and phosphate on coronary atherosclerosis detected by cardiac computed tomography Eur Heart J 2012 33 22 2873 2881 10.1093/eurheartj/ehs152 22719023

[B25] Gutierrez OM Mannstadt M Isakova T Rauh-Hain JA Tamez H Shah A Smith K Lee H Thadhani R Juppner H Fibroblast growth factor 23 and mortality among patients undergoing hemodialysis N Engl J Med 2008 359 6 584 592 10.1056/NEJMoa0706130 18687639 PMC2890264

[B26] Isakova T Xie H Yang W Xie D Anderson AH Scialla J Wahl P Gutierrez OM Steigerwalt S He J Fibroblast growth factor 23 and risks of mortality and end-stage renal disease in patients with chronic kidney disease JAMA 2011 305 23 2432 2439 10.1001/jama.2011.826 21673295 PMC3124770

[B27] Kovesdy CP Ahmadzadeh S Anderson JE Kalantar-Zadeh K Secondary hyperparathyroidism is associated with higher mortality in men with moderate to severe chronic kidney disease Kidney Int 2008 73 11 1296 1302 10.1038/ki.2008.64 18337714

[B28] Fliser D Kollerits B Neyer U Ankerst DP Lhotta K Lingenhel A Ritz E Kronenberg F Kuen E Konig P Fibroblast growth factor 23 (FGF23) predicts progression of chronic kidney disease: the Mild to Moderate Kidney Disease (MMKD) Study J Am Soc Nephrol 2007 18 9 2600 2608 10.1681/ASN.2006080936 17656479

[B29] Gutierrez OM Januzzi JL Isakova T Laliberte K Smith K Collerone G Sarwar A Hoffmann U Coglianese E Christenson R Fibroblast growth factor 23 and left ventricular hypertrophy in chronic kidney disease Circulation 2009 119 19 2545 2552 10.1161/CIRCULATIONAHA.108.844506 19414634 PMC2740903

[B30] Parker BD Schurgers LJ Brandenburg VM Christenson RH Vermeer C Ketteler M Shlipak MG Whooley MA Ix JH The associations of fibroblast growth factor 23 and uncarboxylated matrix Gla protein with mortality in coronary artery disease: the Heart and Soul Study Ann Intern Med 2010 152 10 640 648 20479029 10.1059/0003-4819-152-10-201005180-00004 PMC3079370

[B31] Deo R Katz R Shlipak MG Sotoodehnia N Psaty BM Sarnak MJ Fried LF Chonchol M de Boer IH Enquobahrie D Vitamin D, parathyroid hormone, and sudden cardiac death: results from the Cardiovascular Health Study Hypertension 2011 58 6 1021 1028 10.1161/HYPERTENSIONAHA.111.179135 22068871 PMC3337033

[B32] Morfis L Smerdely P Howes LG Relationship between serum parathyroid hormone levels in the elderly and 24 h ambulatory blood pressures J Hypertens 1997 15 11 1271 1276 10.1097/00004872-199715110-00011 9383176

[B33] Rubin MR Maurer MS McMahon DJ Bilezikian JP Silverberg SJ Arterial stiffness in mild primary hyperparathyroidism J Clin Endocrinol Metab 2005 90 6 3326 3330 10.1210/jc.2004-1400 15769995

[B34] Tomaschitz A Ritz E Pieske B Fahrleitner-Pammer A Kienreich K Horina JH Drechsler C Marz W Ofner M Pieber TR Aldosterone and parathyroid hormone: a precarious couple for cardiovascular disease Cardiovasc Res 2012 94 1 10 19 10.1093/cvr/cvs092 22334595

[B35] Mehrotra R Kermah DA Salusky IB Wolf MS Thadhani RI Chiu YW Martins D Adler SG Norris KC Chronic kidney disease, hypovitaminosis D, and mortality in the United States Kidney Int 2009 76 9 977 983 10.1038/ki.2009.288 19657329 PMC3791220

[B36] Navaneethan SD Schold JD Arrigain S Jolly SE Jain A Schreiber MJ Jr Simon JF Srinivas TR Nally JV Jr Low 25-hydroxyvitamin D levels and mortality in non-dialysis-dependent CKD Am J Kidney Dis 2011 58 4 536 543 10.1053/j.ajkd.2011.04.028 21816525 PMC3199572

[B37] Ravani P Malberti F Tripepi G Pecchini P Cutrupi S Pizzini P Mallamaci F Zoccali C Vitamin D levels and patient outcome in chronic kidney disease Kidney Int 2009 75 1 88 95 10.1038/ki.2008.501 18843258

